# Evolution of Plant HECT Ubiquitin Ligases

**DOI:** 10.1371/journal.pone.0068536

**Published:** 2013-07-15

**Authors:** Ignacio Marín

**Affiliations:** Instituto de Biomedicina de Valencia-Consejo Superior de Investigaciones Científicas (IBV-CSIC), Valencia, Spain; Beijing Institute of Genomics, Chinese Academy of Sciences, China

## Abstract

HECT ubiquitin ligases are key components of the ubiquitin-proteasome system, which is present in all eukaryotes. In this study, the patterns of emergence of HECT genes in plants are described. Phylogenetic and structural data indicate that viridiplantae have six main HECT subfamilies, which arose before the split that separated green algae from the rest of plants. It is estimated that the common ancestor of all plants contained seven HECT genes. Contrary to what happened in animals, the number of HECT genes has been kept quite constant in all lineages, both in chlorophyta and streptophyta, although evolutionary recent duplications are found in some species. Several of the genes found in plants may have originated very early in eukaryotic evolution, given that they have clear similarities, both in sequence and structure, to animal genes. Finally, in *Arabidopsis thaliana*, we found significant correlations in the expression patterns of HECT genes and some ancient, broadly expressed genes that belong to a different ubiquitin ligase family, called RBR. These results are discussed in the context of the evolution of the gene families required for ubiquitination in plants.

## Introduction

Ubiquitination is involved in multiple essential functions in all eukaryotes. First, it has a critical role in the regulation of protein levels, given that the addition of a polyubiquitin chain often targets a protein for proteasomal degradation. In addition, ubiquitination has other important tasks which often do not require the degradation of the tagged proteins. This versatility explains why many cellular processes are controlled by the ubiquitination machinery [1]–[6]. Given its wide functional implications, there is a great interest in understanding in detail the families of proteins which constitute the ubiquitination system. Among them, the most diverse components are the ubiquitin ligases (E3s), the group of enzymes able to transfer ubiquitin to target proteins, which provide specificity to the ubiquitination machinery. The genes encoding these enzymes, often very numerous, are classified into several classes. This classification depends on two characteristics: 1) whether they are single proteins or members of multiprotein complexes, and 2) their structural and functional features [1]. In recent studies, we have analyzed the evolution of several types of ubiquitin ligases, such as RING finger-containing E3s (RBR and TRIM families [7]–[12]), cullin-containing E3 complexes [13], U-box E3s [14] and HECT E3s [15].

HECT E3s are one of the main classes of ubiquitin ligases. They are characterized by having a C-terminal HECT domain, involved in both accepting ubiquitin from an ubiquitin-conjugating protein and catalyzing its transfer to the protein to be ubiquitinated [16]. It has been also shown that a few mammalian HECT proteins may attach the ubiquitin-like protein ISG15, instead of ubiquitin, to its substrates [17]–[19]. The functions of animal HECTs have been studied in detail. They have critical roles regulating several basic cellular mechanisms such as signal transduction pathways, protein trafficking or DNA damage. Mutations in human HECT genes are involved in the genesis of several diseases [16], [20]–[23].

The presence of a HECT domain is exclusive of HECT E3s. Therefore, it is very simple to establish whether a particular protein belongs to this family. In addition, the HECT protein domain is long enough (about 350 amino acids) as to provide significant information for phylogenetic analyses. These two facts together allow for precise studies of the origin and evolution of HECT-encoding genes. In one of our previous works, the evolution of animal and choanoflagellate HECT ubiquitin ligases was analyzed in great detail [15]. It was determined that in animals there are 16 HECT subfamilies, composed by proteins with very similar sequences that also often have subfamily-specific protein domains. 14 of these subfamilies originated either before the origin of animals (i. e. they are present in both animals and choanoflagellates) or very early in animal evolution, while the other two are chordate-specific [15]. This pattern means that HECT family diversification mostly occurred before the emergence of some of the key animal-specific signal transduction systems that are regulated by HECT proteins. It was also determined that, after the expansion of the family at the origin of animals, several lineages (e. g. insects, nematodes, urochordates), have lost a substantial number of HECT genes, while a considerable increase by gene duplication has occurred in a single lineage, vertebrates [15]. These results were strikingly similar to those found for the RBR family of ubiquitin ligases [10].

Plant HECTs have not been studied in detail. The only plant species for which HECTs have been hitherto analyzed is *Arabidopsis thaliana*. This species contains seven HECT genes (called *UPL1* - *UPL7*). The proteins encoded by the *UPL* genes were classified into four subfamilies according to both HECT domain sequence similarity and protein structure [24]. Proteins similar to three of those subfamilies were detected in both animals and fungi, suggesting that they emerged in early eukaryotic evolution [24], [25]. It is difficult however to compare these results with the more comprehensive analyses performed in animals, given the lack of a detailed study of the patterns of diversification of HECTs in other plants. In this study, a complete characterization of the evolution of HECT genes in green algae and higher plants is performed, to determine their early evolution and their patterns of duplication in plant lineages. These results allow for a precise comparison of the evolution of plant and animal HECTs, as well as a characterization of the similarities and differences in the evolutionary patterns of several ubiquitin ligase families in viridiplantae.

## Results

### Diversification of HECT Ubiquitin Ligases in Plant Lineages

A comprehensive database with 413 HECT domain sequences derived from viridiplantae species was generated (see Methods). These sequences belonged to 1) chlorophytes (55 sequences from 9 different species); 2) basal streptophytes (from the genera *Chlorokybus* [which belongs to the Chlorokybophyceae], *Klebsormidium* [Klebsormidiophyceae], *Penium* [Zygnemophyceae], *Coleochaetae* [Streptophytina, Coleochaetophyceae], *Nitella* [Streptophytina; Charophyceae], *Pellia* [Streptophytina, Embryophyta, Marchantiophyta], *Physcomitrella* [Streptophytina, Embryophyta, Bryophyta] and *Selaginella* [Streptophytina, Embryophyta, Tracheophyta, Lycopodiophyta]; a total of 41 sequences from 9 species); and, 3) spermatophytes (gymnosperms: 9 sequences from 3 species; angiosperms: 303 sequences from 64 different species).

**Figure 1:**
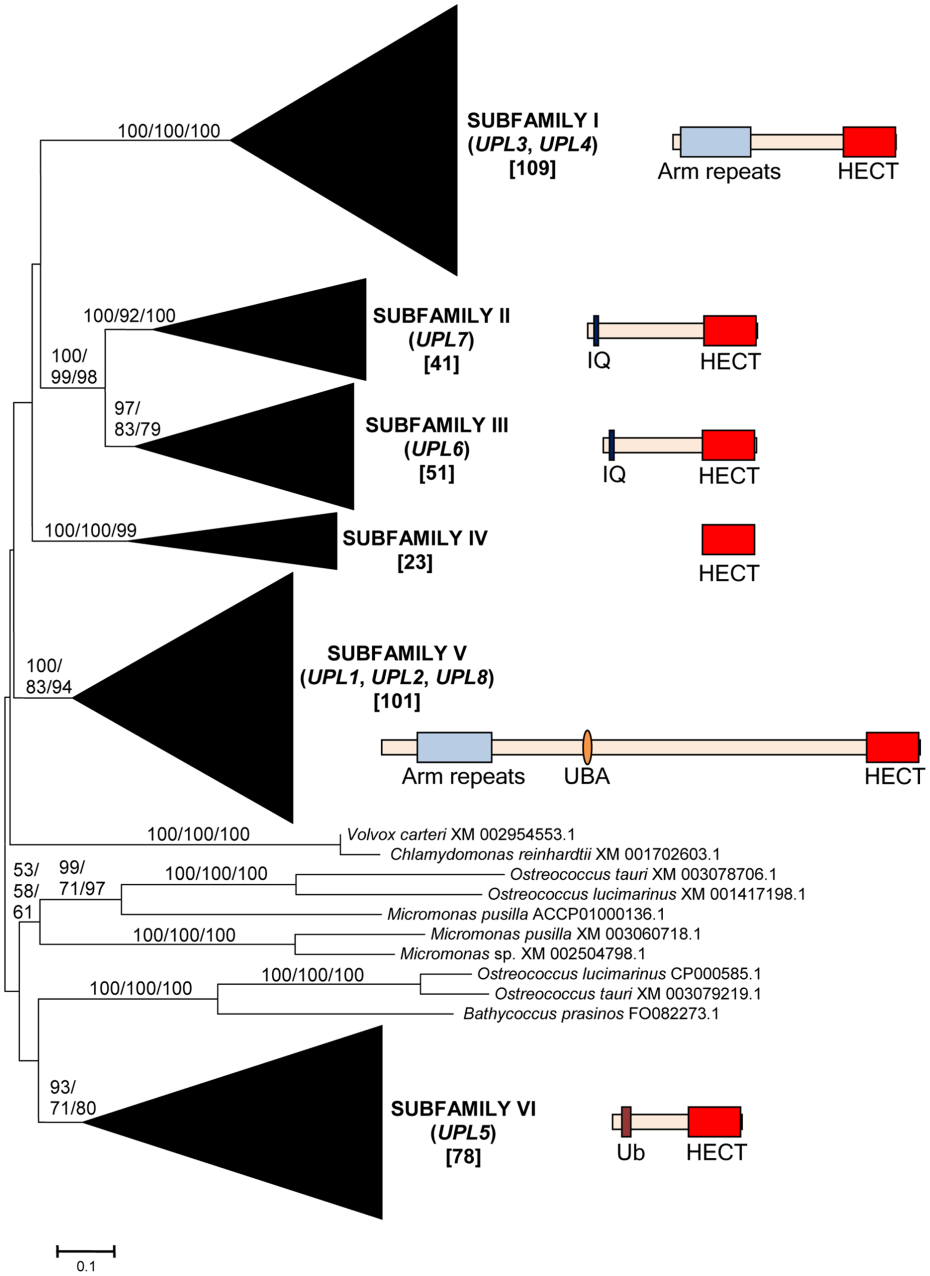
Basic result for the phylogenetic analysis including 413 plant HECT sequences. The main branches that correspond to the six subfamilies (I – VI) are indicated. Only a few green algal sequences were excluded from those branches. Numbers above those branches correspond to bootstrap support, in percentages. The three numbers correspond to Neighbor-joining (NJ), Maximum Parsimony (MP) and Maximum Likelihood (ML) analyses (order: NJ/MP/ML). The names of the angiosperm genes found in each family (*UPL1*-*UPL8*) are also indicated. Subfamily IV is not present in angiosperms (see main text). Numbers in brackets refer to the number of protein sequences which are included in each branch. Only branches with bootstrap support above 50% in all three analyses are indicated. The structures typical of proteins of the different subfamilies are also indicated. In addition to the C-terminal HECT domains (red boxes), other domains can be found, as armadillo repeats (Arm repeats; in Subfamilies I and V), IQ domains (in Subfamilies II and III), UBA domains (Subfamily V) or ubiquitin domains (Ub; Subfamily VI). Proteins are drawn at scale, with the HECT domain corresponding to 350 amino acids.

**Figure 2:**
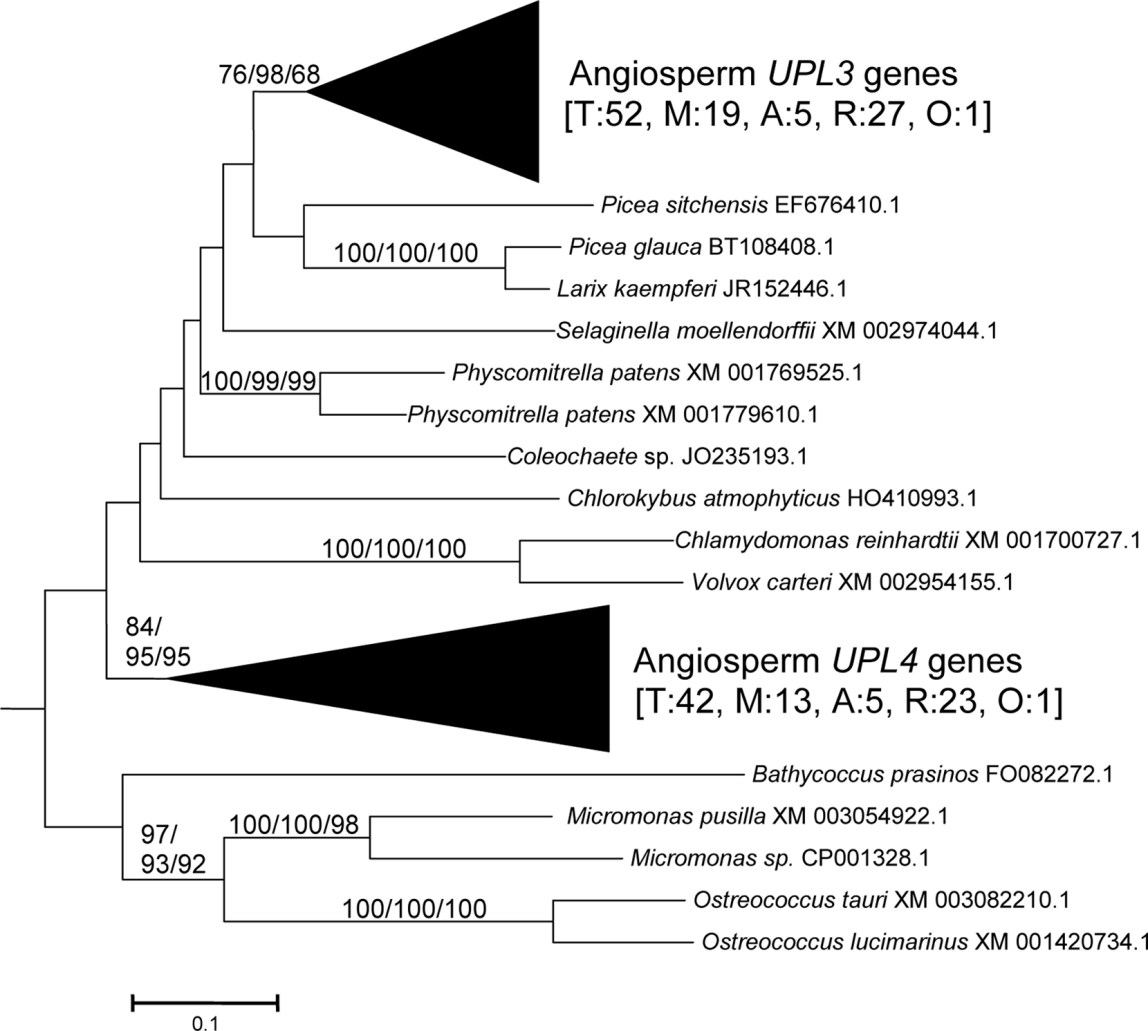
Subfamily I sequences. Angiosperm sequences are named accordingto the *Arabidopsis* genes (*UPL3* and *UPL4*). Bootstrap support and number of sequences are indicated as in Figure 1. The numbers in brackets indicate first the total number of sequences (T) and then the number of sequences in monocots (M), asterid dicots (A), rosid dicots (R), or other dicots not included in those two groups (Other: O).

**Figure 3:**
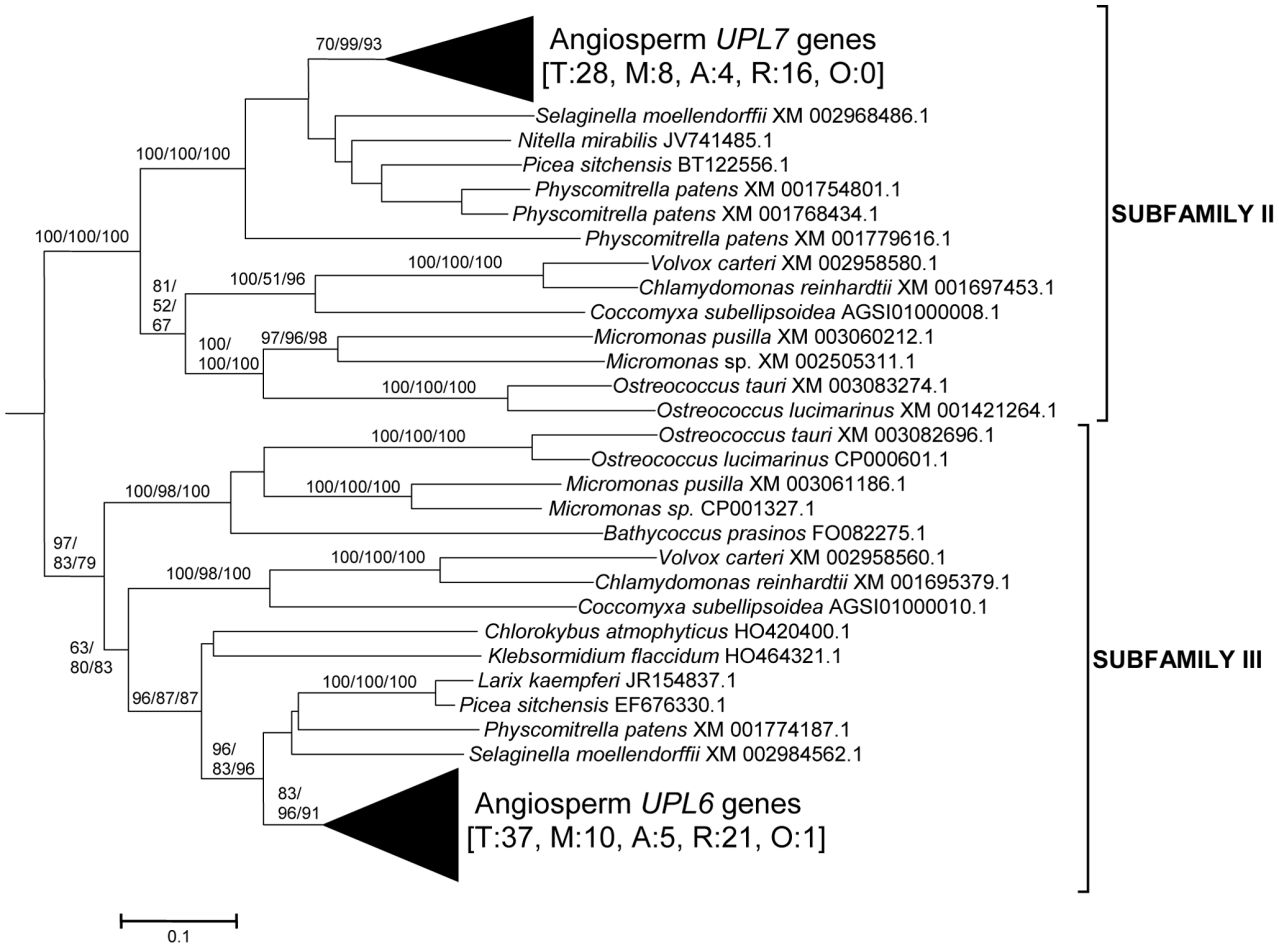
Sequences corresponding to Subfamilies II and III. The angiosperm genes *UPL7* and *UPL6*, which respectively belong to Subfamily II and Subfamily III, are indicated. Bootstrap support and number of sequences indicated as in Figure 2.

**Figure 4:**
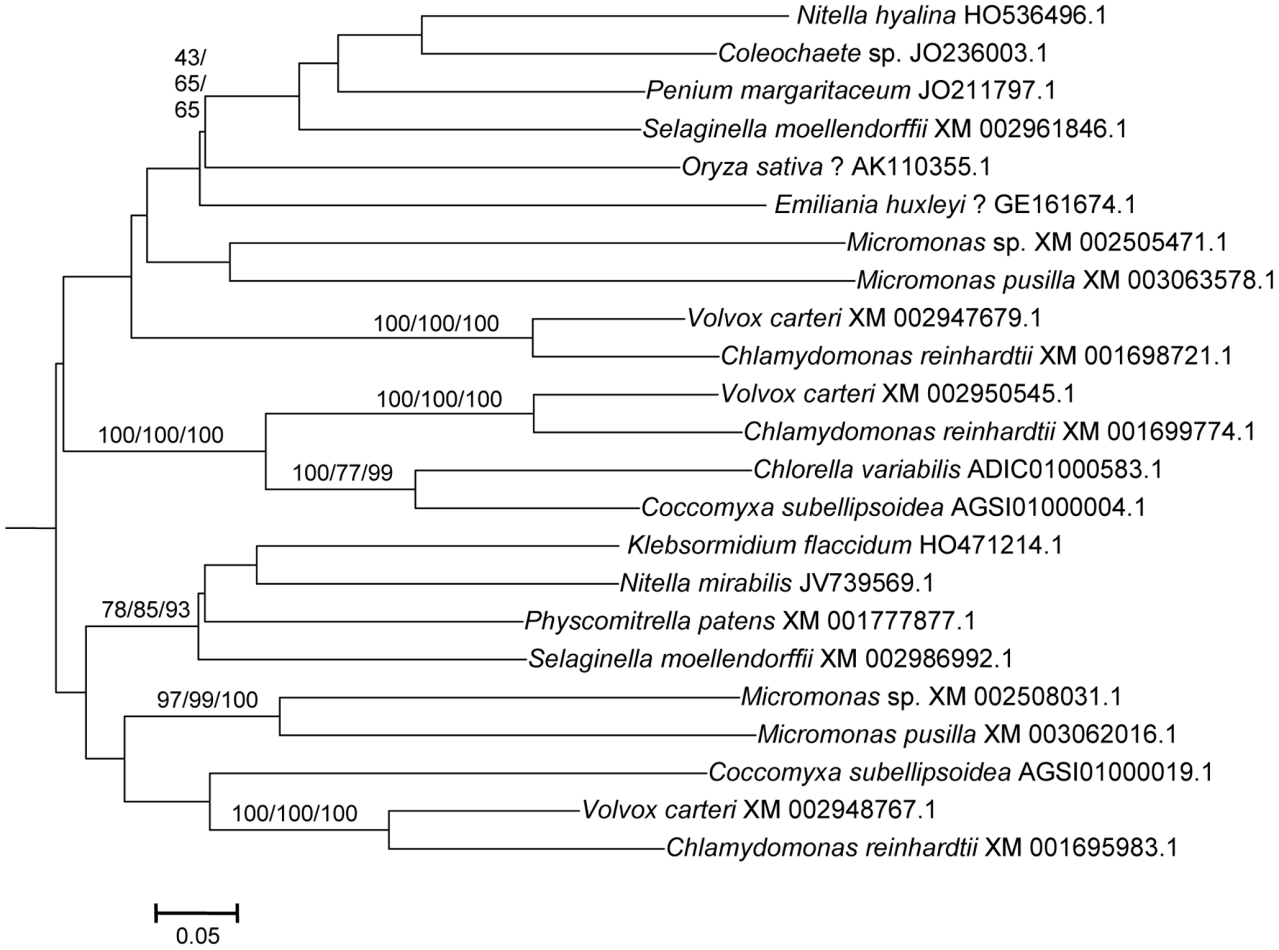
Subfamily IV sequences. Notice the low bootstrap values for many internal branches (see text). The question marks indicate two incongruent results, corresponding to two ESTs that most likely did not come from the species to which they were adscribed (see main text).

**Figure 5:**
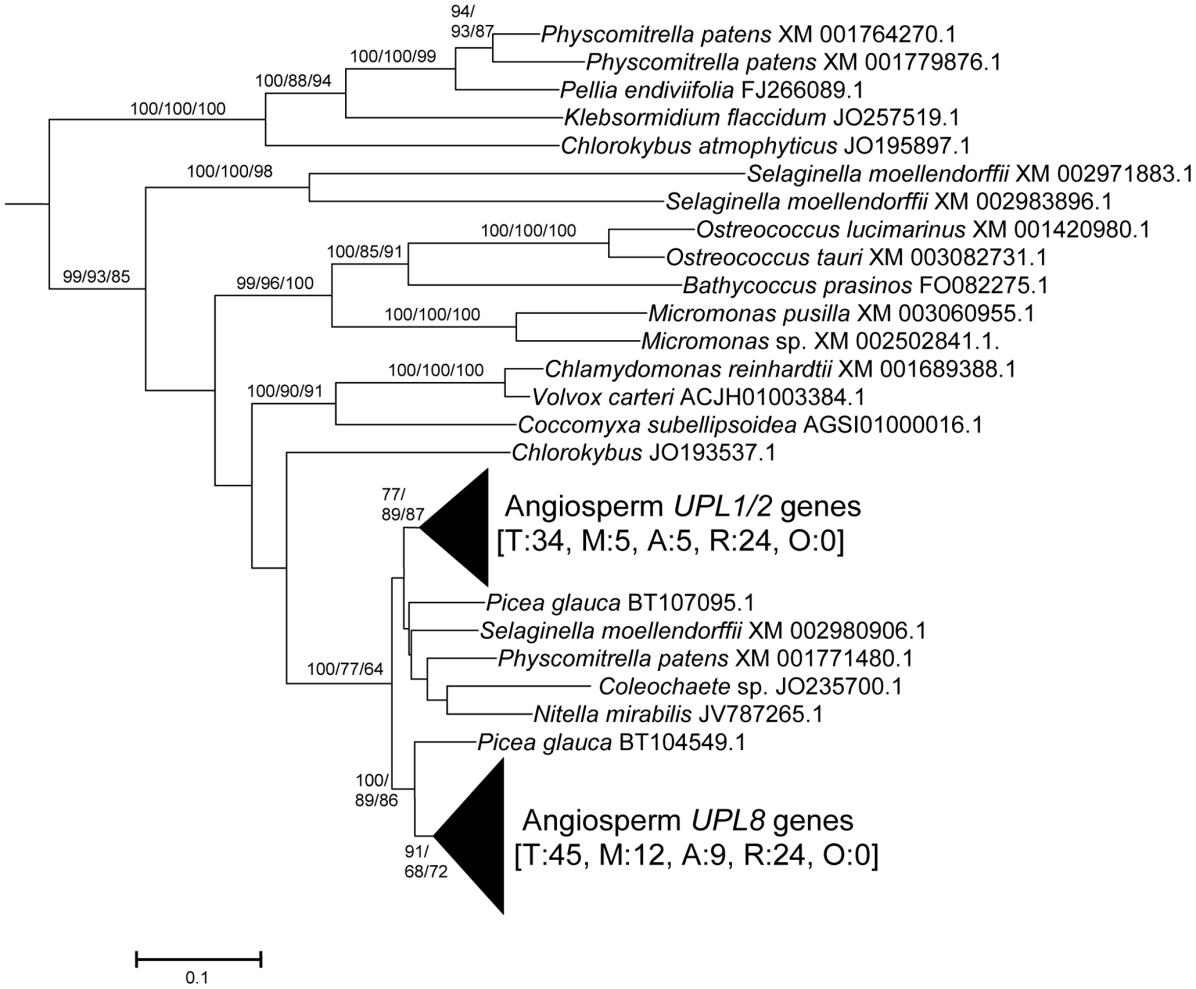
Subfamily V sequences. They include the angiosperm genes *UPL1/2* (from which derive the *A. thaliana* recent duplicates *UPL1* and *UPL2*) and *UPL8*, a new gene, described here for the first time, given that it is absent in *A. thaliana* (see text). Boostrap values and number of sequences as in Figures 2 and 3.

**Figure 6:**
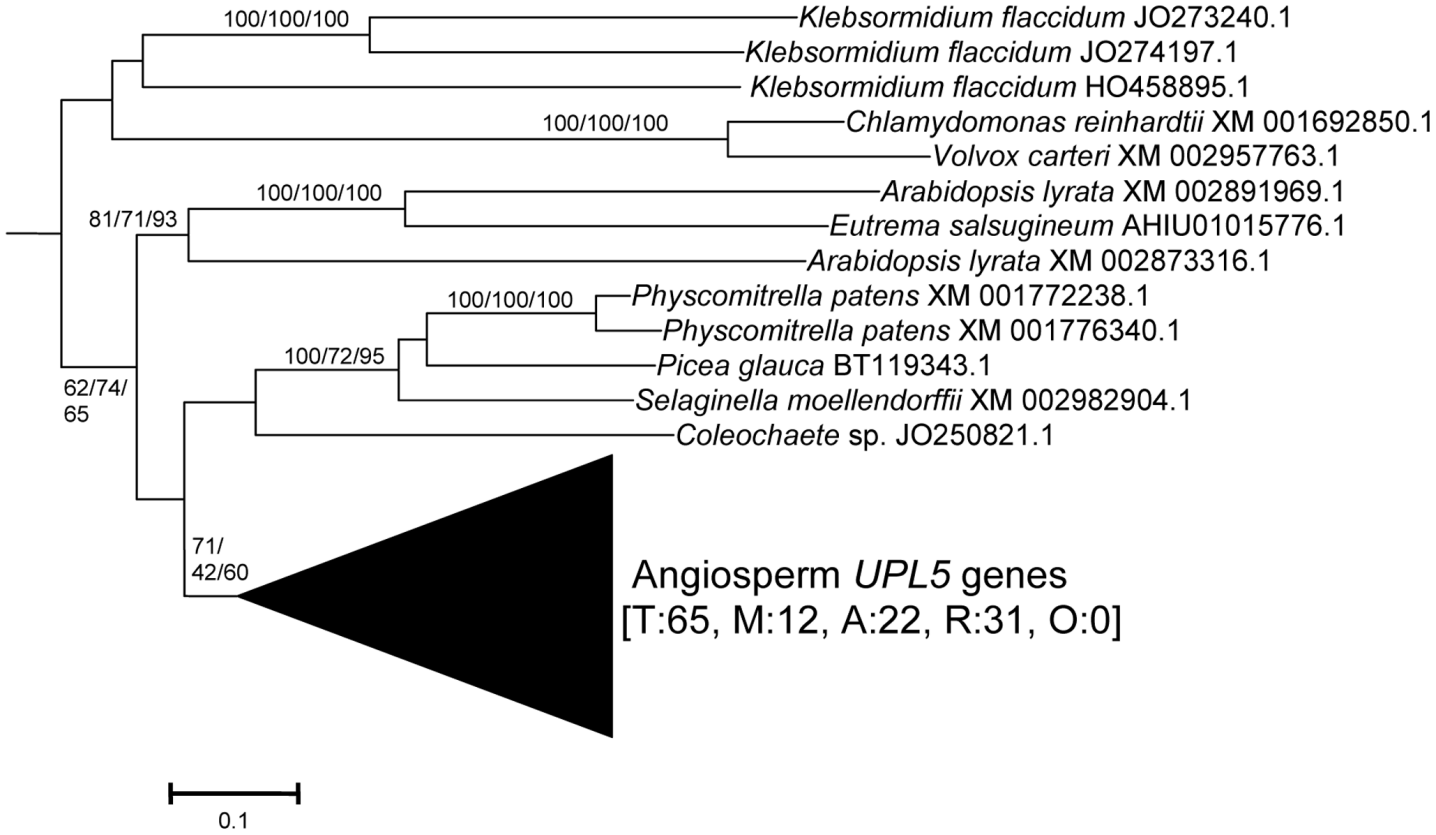
Subfamily VI sequences. This subfamily includes the angiosperm *UPL5* gene. Bootstrap values and number of sequences indicated as in previous figures, i. e. total (T), monocot (M), dicot rosid (R), dicot asterid (A) and dicot, others (O).

From these sequences, the fundamental divisions of HECT E3s in plants were characterized. Figures 1, 2, 3, 4, 5, 6 summarize the main results. Phylogenetic analyses demonstrated that plant HECTs can be classified into six main subfamilies, named I to VI in this study (Figure 1). The ancient origin of these subfamilies is supported by members of all them being found both in green algae and in higher plants. With the exception of Subfamily IV, each of them is not only characterized by all proteins having very similar HECT domain sequences, but also by an independent indicator: the presence, in most cases, of characteristic protein domains located N-terminally respect to the HECT domain. Figures 2, 3, 4, 5, 6, which correspond to expanded sections of the compact tree presented in Figure 1, describe in more detail the phylogenetic results for the different subfamilies. In those figures, the *Arabidopsis* names for the HECT genes (*UPL1-7*) are used to indicate not only those particular genes, but also their orthologs present in other angiosperms. A summary of the main results is as follows:

- Subfamily I: Most proteins in this family contain an N-terminal Armadillo repeat region (Figure 1), although, both in this and in the following subfamilies, some truncated proteins lacking that domain were detected. Also, a single, exceptional protein which has a CCHC zinc finger instead of the Armadillo repeat was detected in *Medicago truncatula* (Accession no. XM_003625529.1). Typically, there is a single Subfamily I gene in chlorophytes, bryophytes, lycophytes and gymnosperms and two in angiosperm species. In *Arabidopsis thaliana*, those two genes are *UPL3* (also known as *Kaktus*) and *UPL4* (also called *Kli5*). The high similarity of those two genes was already noticed in previous works [24], [25].
- Results for subfamilies II and III are summarized together in Figure 3, given that the global phylogenetic analyses (as shown in Figure 1) demonstrated that they are closely related. A single gene of each subfamily is present in all main plant lineages, although some species-specific duplicates have been detected. Characteristic of most members of both subfamilies is the presence of an IQ domain (Figure 1). The two *Arabidopsis* genes *UPL7* and *UPL6* are respectively members of Subfamilies II and III. The close similarity of those two genes was already detected in [24], [25]. However, the fact that two different paralogous genes can be found both in green algae and in higher plants indicates that it is better to establish two different subfamilies than to lump together all those genes into a single subfamily, as suggested before [24].
- Subfamily IV is novel family, which had not been hitherto described, given that it is absent in *Arabidopsis*. It is the only one for which phylogenetic results are unclear (Figure 4). Subfamily IV proteins are very short (Figure 1), and the general lack of any characteristic additional protein domain further complicates understanding their relationships. Only two *Micromonas* species have genes (Accession numbers XM_003062016.1 and XM_002508031.1) that encode proteins with RCC1 repeats, but this is clearly a recent acquisition. Green algae typically have 2–3 genes of this subfamily and two main lineages seem to be present in some streptophyta (*Nitella*, *Selaginella*), although bootstrap support is low. Therefore, the simplest hypothesis that can be formulated with the available data is that two Subfamily IV genes existed before the split that separated green algae from the rest of plants. However, other explanations, based on independent duplications, cannot be disregarded at present. Notice also that Figure 4 shows two results that are phylogenetically incongruent (indicated in the figure with a question mark). First, a single spermatophyte sequence detected derived an EST supposedly derived from *Oryza sativa*. The fact that none of the *Oryza* genome projects found this sequence, as well as the absence of Subfamily IV genes in all other angiosperms, indicates that it must belong to some other species. Also, a second EST, supposedly derived from the haptophyte *Emiliania huxleyi*, actually has such a great similarity to typical plant sequences that it must be another incorrectly ascribed sequence.
- Subfamily V results (Figure 5) indicate that a single gene was present before the green algae separated from the rest of plants. After that, the simplest explanation of the pattern observed requires two independent duplications. The first one occurred in the very early evolution of the streptophytes. Later, one of these duplicated genes was lost in spermatophytes. The second duplication occurred just before the gymnosperm/angiosperm split, generating two genes which I have called *UPL1*/2 and *UPL8*. These names reflect the relationship of these genes with the ones present in *Arabidopsis thaliana*. It turns out that the situation found in *A. thaliana* is exceptional. The two very similar *A. thaliana* genes of Subfamily V (*UPL1* and *UPL2*) were generated by a very recent duplication of the *UPL1/2* gene (hence this name, meaning that it is the ancestor of both *Arabidopsis* genes *UPL1* and *UPL2*). This is demonstrated by the fact that a single *UPL1/2* gene is present in other brassicaceae species. In addition, the other Subfamily V gene present in most spermatophytes, which called here for the first time *UPL8*, had never been described given that it has been lost in *A. thaliana* (although is present in other brassicaceae, including *Arabidopsis lyrata*). Most subfamily V genes, including the angiosperm genes *UPL1/2* and *UPL8*, encode proteins that contain armadillo repeats and an UBA domain, in addition to the HECT domain (see Figure 1).
- Finally, Subfamily VI has a simple history, with a single gene present in all species, plus some species-specific duplicates (e. g. in *Arabidopsis lyrata*, *Physcomytrella*, *Klebsormidium*). These genes typically encode proteins with an additional ubiquitin domain (indicated in Figure 1). *UPL5* is the only *Arabidopsis thaliana* gene that belongs to this Subfamily.

**Figure 7:**
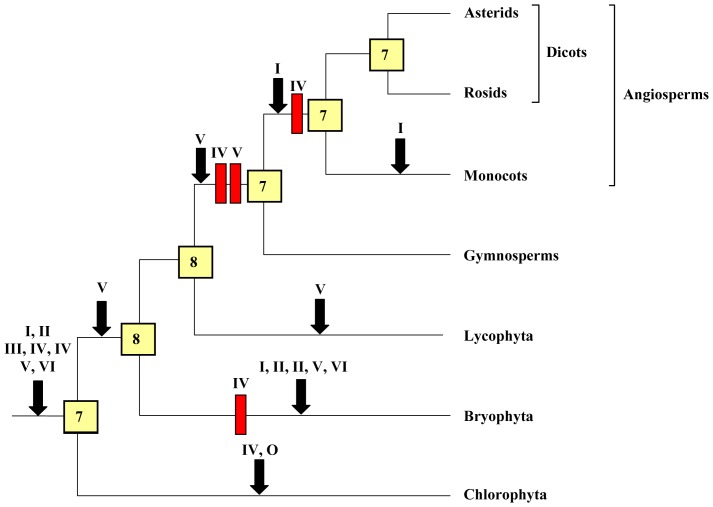
The most parsimonious hypothesis to explain the evolution of HECT genes in plants. Red rectangles correspond to gene losses and black arrows to gene emergences. Subfamilies are indicated with roman numerals; O means “other”, indicating the presence of an additional gene in green algae (see Figure 1). The numbers in the boxes correspond to the genes deduced to exist in the ancestors of the corresponding lineages. The loss of a Subfamily IV gene in angiosperms is supported by a single fragment of a putative gymnosperm Subfamily IV gene (see text), so it must be considered a provisional result, until additional sequences are available.

These results show that the evolution of HECT ubiquitin ligases in plants has been in general extremely conservative: large gene amplifications are totally absent. Figure 7 summarizes the most parsimonious hypothesis that explains the results observed for the main viridiplantae lineages for which extensive genomic data are available. This figure summarizes not only the sequences included in Figures 1, 2, 3, 4, 5, 6, but also some additional data corresponding to HECT sequences which were not included in the original dataset given that they are truncated, partial ones. These additional sequences were found in specific searches focused on taxa for which the number of full-length sequences is low (see Methods). Of particular importance was the finding of fragments of Subfamily IV genes in the gymnosperm *Pinus taeda* (accession numbers DR058599.1 and DR116961.1), which indicate that at least one gene of this subfamily is present in gymnosperms. Additional significant fragments of Subfamily IV sequences were found in *Marchantia polymorpha* [Streptophytina, Embryophyta, Marchantiophyta; accession numbers BJ846038.1, BJ866343.1, BJ871837.1]. However, none was found in angiosperms, confirming the results already indicated above.

Figure 7 summarizes the fact that the basic number of genes present in the ancestors of all lineages examined has been almost constant (7–8) along hundreds of millions of years, in spite of the multiple genome duplications that occurred in higher plans. Table 1 summarizes the exact results for some model species. The numbers vary from 5 to 14 genes, due to some independent, recent, lineage-specific losses or duplications. However, significantly, most living model species have 6–9 genes, a number very similar to that determined for their ancestors.

**Table 1:** Number of HECT genes in selected species.

SPECIES	Taxonomic group	I	II	III	IV	V	VI	Others	Total
*Chlamydomonas reinhardtii*	Green algae	1	1	1	3	1	1	1	9
*Ostreococcus tauri*	Green algae	1	1	1	0	1	0	2	6
*Micromonas pusilla*	Green algae	1	1	1	2	1	0	2	8
*Physcomitrella patens*	Bryophytes	2	3	1	1	3	2	0	12
*Selaginella moellendorffii*	Lycophytes	1	1	1	2	3	1	0	9
*Picea sitchensis*	Gymnosperms	1	1	1	1	1	0	0	5
*Hordeum vulgare*	Angiosperms, monocots	1	1	1	0	1	1	0	5
*Zea mays*	Angiosperms, monocots	4	1	1	0	2	1	0	9
*Sorghum bicolor*	Angiosperms, monocots	4	1	1	0	2	1	0	9
*Oryza sativa*	Angiosperms, monocots	3	1	1	1?	2	1	0	8–9
*Solanum tuberosum*	Angiosperms, dicots, asterids	2	1	1	0	2	6	0	12
*Vitis vinifera*	Angiosperms, dicots, rosids	3	1	1	0	2	1	0	8
*Glycine max*	Angiosperms, dicots, rosids	6	1	3	0	2	2	0	14
*Populus trichocarpa*	Angiosperms, dicots, rosids	2	1	2	0	3	1	0	9
*Arabidopsis thaliana*	Angiosperms, dicots, rosids	2	1	1	0	2	1	0	7

I-VI refer to the six HECT subfamilies. The column marked as “Others” includes the few sequences shown in Figure 1 that cannot be included in any subfamily. The question mark indicates a gene that is most likely falsely attributed to *Oryza sativa* (see text).

### Comparison of Plant and Animal HECT Genes

It has been described in the previous section that most plant HECT subfamilies are defined not only by the high sequence similarity of the HECT domains present in their members but also by structural features, given that most members of each subfamily often contain characteristic protein domains. In principle, it should be possible to use all that information to trace back in time the evolutionary history of HECT proteins. Whether there are other lineages, distantly related to viridiplantae, with the same subfamilies could be demonstrated if those lineages contained proteins with similar HECT domain sequences and, at the same time, with the same structural features that those found in plants. Actually, some preliminary evidence for the presence of ancient lineages of HECT proteins was already described [24], [25]. The more precise classification for animal HECTs recently obtained [15] together with the data presented in this study should allow for a much more precise characterization of the relationships of all HECT subfamilies in these organisms.

**Figure 8:**
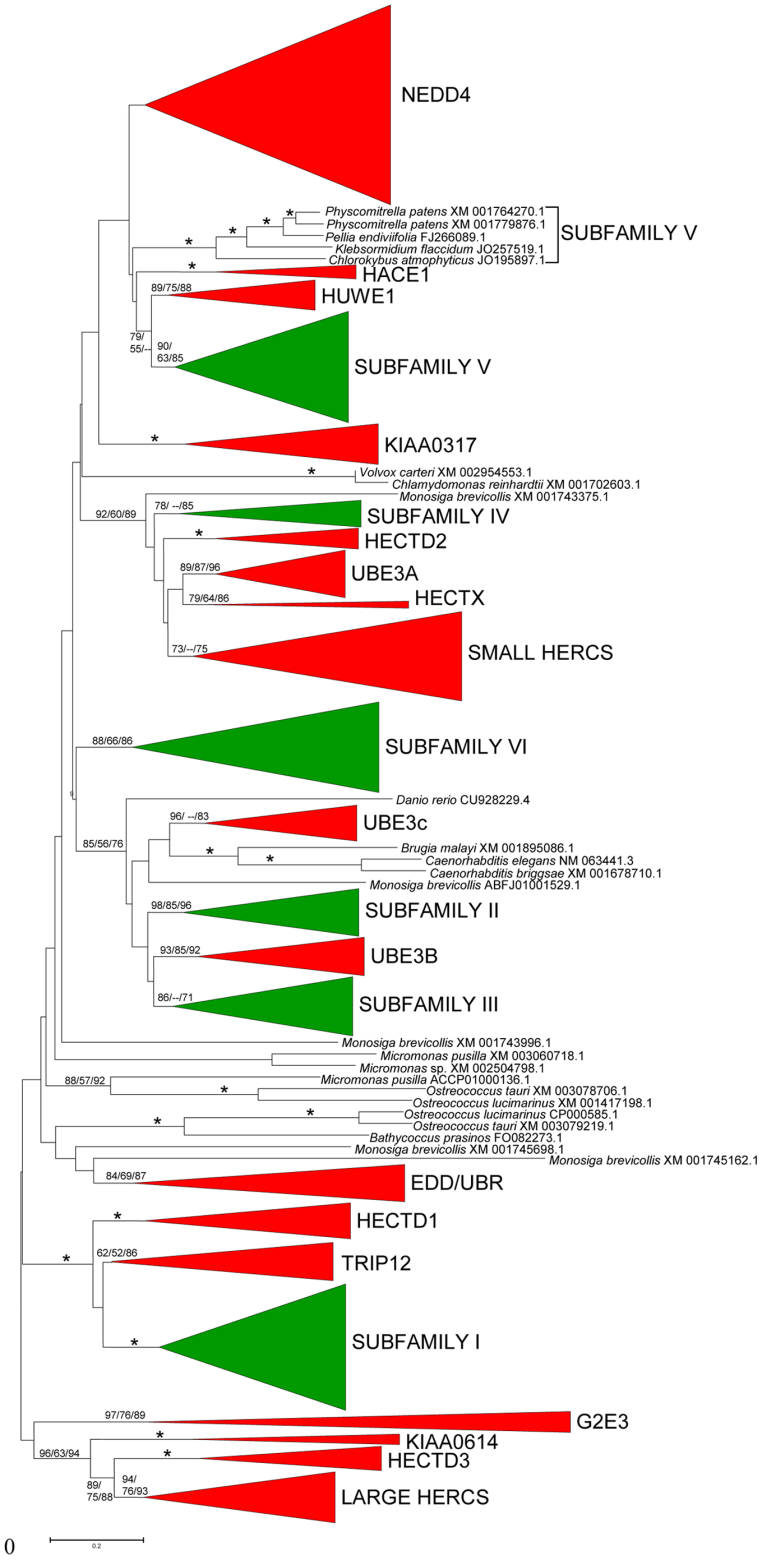
Phylogenetic tree comparing plant (green) and animal (red) HECT subfamilies. Bootstrap values for the most relevant branches are indicated (again as NJ/MP/ML). Asterisk indicate branches for which the three phylogenetic analyses provided values higher than 90%. Only a few sequences cannot be ascribed to the main subfamilies.

Figure 8 summarizes the results of the comparison of plant and animal HECTs. Although the bootstrap support is in general not very high, the results are compatible with all plant subfamilies except Subfamily VI having counterparts in animals. The most similar animal subfamilies are respectively TRIP12 (for plant Subfamily I), the UBE3B/3C subfamily (for plant subfamilies II and III), a monophyletic ensemble of animal subfamilies described in our previous study [15], which is composed by the HECTD2, UBE3A/E6-AP, HECTX and the SMALL HERCs subfamilies (for plant subfamily IV) and HUWE1 (for plant subfamily V) (Figure 8). It was known already that all these animal HECT subfamilies potentially related to the plant ones were ancient, emerging before the origin of animals [15]. The putative relationships deduced from the results in Figure 8 are strengthened by the fact that the protein structures of the animal and plant subfamilies are compatible in all cases. Thus, both TRIP12 and Subfamily I proteins contain armadillo domains, IQ domains are present in both the animal UBE3B/3C subfamily and plant subfamilies II and III and both HUWE1 and plant Subfamily V proteins have UBA domains (see [15] and data above). Also, neither plant subfamily IV nor the corresponding animal proteins (with the exception of the SMALL HERC subfamily proteins, which recently acquired RCC1 repeats [15]) have additional protein domains. Given that the acquisition of protein domains is a rare event, this congruence in both sequence similarity and structure indicates that four different types of proteins existed before the plant/animal split, thus emerging very early in eukaryotic evolution. Preliminary evidence suggests that these four HECT groups are present in fungi and some proteins with related structures and similar HECT domain sequences can also be detected in several other protist groups, such as excavates or alveolates (unpublished results). In summary, it seems very likely that multiple ubiquitin ligases of the HECT family already existed in the last eukaryotic common ancestor. Related results have been obtained in a recent work [26]. However, some significant discrepancies can be detected when their results are compared with those shown in this study. For example, and just focusing on plant genes, they were unable to detect Subfamily IV and missed the existence of *UPL8* genes. The very limited number of green algae and plant species that they analyzed (a total of six, including just one angiosperm, *A. thaliana*) explains these differences.

### Patterns of Expression of HECT Genes in *Arabidopsis*

**Figure 9:**
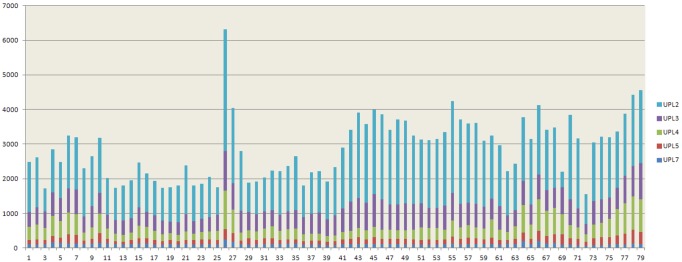
Cumulative values of expression for *Arabidopsis* HECT genes in 79 developmental samples. Data were obtained from Schmid *et al.* [53]. The Y-axis is measured in arbitrary expression units. Samples are as follows: 1) root 7 days; 2) root 17 days; 3) root 15 days; 4) root 8 days; 5) root 8 days; 6) root 21 days; 7) root 21 days; 8) stem: hypocotyl; 9) stem: first node; 10) stem: second internode; 11) cotyledons; 12) leaves 1+2; 13) rosette leaf #4, 1 cm long; 14) rosette leaf #4, 1 cm long (gl1-T mutant); 15) rosette leaf # 2; 16) rosette leaf # 4; 17) rosette leaf # 6; 18) rosette leaf # 8; 19) rosette leaf # 10; 20) rosette leaf # 12; 21) rosette leaf # 12 (gl1-T mutant); 22) leaf 7, petiole; 23) leaf 7, petiole; 24) leaf 7, distal half; 25) leaf, 15 days; 26) leaf, senescing; 27) cauline leaves; 28) seedling, green parts, 7 days; 29) seedling, green parts, 8 days; 30) seedling, green parts, 8 days; 31) seedling, green parts, 21 days; 32) seedling, green parts, 21 days; 33) whole plant: developmental drift, entire rosette after transition to flowering, but before bolting, 21 days; 34) whole plant: developmental drift, entire rosette after transition to flowering, but before bolting, 22 days; 35) whole plant: developmental drift, entire rosette after transition to flowering, but before bolting, 23 days; 36) vegetative rosette 7 days; 37) vegetative rosette 14 days; 38) vegetative rosette 21 days; 39) shoot apex, vegetative+young leaves; 40) shoot apex, vegetative; 41) shoot apex, transition (before bolting); 42) shoot apex, inflorescence (after bolting); 43) shoot apex, inflorescence (after bolting) (clv3-7 mutant); 44) shoot apex, inflorescence (after bolting) (lfy-12 mutant); 45) shoot apex, inflorescence (after bolting) (ap1-15 mutant); 46) shoot apex, inflorescence (after bolting) (ap2-6 mutant); 47) shoot apex, inflorescence (after bolting) (ufo-1 mutant); 48) shoot apex, inflorescence (after bolting) (ap3-6 mutant); 49) shoot apex, inflorescence (after bolting) (ag-12 mutant); 50) flowers stage 9; 51) flowers stage 10/11; 52) flowers stage 12; 53) flower stage 12; multi-carpel gynoeceum; enlarged meristem; increased organ number (clv3-7 mutant); 54) flower stage 12; shoot characteristics; most organs leaf-like (lfy-12 mutant); 55) flower stage 12; sepals replaced by leaf-like organs, petals mostly lacking, has secondary flowers (ap1-15 mutant); 56) flower stage 12; no sepals or petals (ap2-6 mutant); 57) flower stage 12; filamentous organs in whorls two and three (ufo-1 mutant); 58) flower stage 12; no petals or stamens (ap3-6 mutant) 59) flower stage 12; no stamens or carpels (ag-12 mutant); 60) flowers stage 15; 61) flowers 28 days; 62) flowers stage 15, pedicels; 63) flowers stage 12, sepals; 64) flowers stage 15, sepals; 65) flowers stage 12, petals; 66) flowers stage 15, petals; 67) flowers stage 12, stamens; 68) flowers stage 15, stamen; 69) mature pollen 70) flowers stage 12, carpels; 71) flowers stage 15, carpels; 72) siliques, w/seeds stage 3; mid globular to early heart embryos; 73) siliques, w/seeds stage 4; early to late heart embryos; 74) siliques, w/seeds stage 5; late heart to mid torpedo embryos; 75) seeds, stage 6, w/o siliques; mid to late torpedo embryos; 76) seeds, stage 7, w/o siliques; late torpedo to early walking-stick embryos; 77) seeds, stage 8, w/o siliques; walking-stick to early curled cotyledons embryos; 78) seeds, stage 9, w/o siliques; curled cotyledons to early green cotyledons embryos; 79) seeds, stage 10, w/o siliques; green cotyledons embryos.

In a previous work, I examined the patterns of expression of RBR ubiquitin ligases, finding that there was a set of genes that were at the same time evolutionary conserved and broadly expressed at high levels, while many others, most of them recently appeared, had very low expression levels in most tissues [11]. Here, the patterns of expression of HECT genes in *Arabidopsis thaliana* were similarly explored. Results from 79 developmental stages were compiled (see Methods), and it was found that data for five of the seven *A. thaliana* HECT genes (*UPL2*, *UPL3*, *UPL4*, *UPL5* and *UPL7*) were available. Results are summarized in Figure 9 and Table 2. The average expression values of all genes were high, ranging from 111.4±3.1 to 1603.4±64.0 expression units. Although expression was quite similar in all tissues, suggesting that these genes may have housekeeping roles, quantitative differences were observed (Figure 9). Actually, a striking resemblance of Figure 2 and the pattern of developmental expression found for the group of broadly expressed RBR genes [11] was detected. If we obtain the average expression for all those RBRs and we compared it with the average for the HECT genes, the Pearson correlation coefficient for the expression values in the 79 tissues is positive and highly significant (r = 0.82, p<10^−7^). Individual comparisons between the RBR and HECT genes established that correlation coefficients were also positive in 44 out of 45 cases and these positive correlations were statistically significant in 29 of those 45 comparisons, after Bonferroni’s correction (Table 2). More precisely, nine RBR genes were tested, and each individual HECT gene significantly correlated with 4 to 8 of them (see also Table 2). The conclusion is that there is a clear similarity in expression patterns in the group of evolutionary conserved and broadly expressed RBR genes described in Ref. [11] and the HECT genes tested here.

**Table 2:** Comparisons of the patterns of expression of housekeeping RBR genes (named in the first column) and HECT genes (*UPL2-7*).

HECT genes	*UPL2* (V)	*UPL3* (I)	*UPL4* (I)	*UPL5* (VI)	*UPL7* (II)
RBR genes *AT4g19670* (II B)	**0.36**	0.27	**0.46**	0.26	**0.80**
*AT3g53690* (II C)	0.09	0.18	**0.50**	**0.40**	**0.47**
*AT5g10370* (HEL)	**0.81**	**0.79**	**0.57**	**0.63**	0.33
*AT1g32340* (ARA54)	0.12	0.18	**0.62**	**0.50**	**0.80**
*AT2g16090* (ARI A)	**0.68**	**0.59**	0.10	0.19	−0.13
*AT4g34370* (ARI A)	0.26	**0.47**	**0.78**	**0.78**	0.29
*AT1g05890* (ARI B)	0.30	**0.55**	**0.74**	**0.72**	**0.65**
*AT2g31510* (ARI B)	**0.72**	**0.67**	**0.77**	**0.71**	**0.46**
*AT5g63760* (ARI B)	0.19	0.23	**0.51**	**0.46**	0.12

In parentheses, the subfamilies to which the genes belong according to Ref. 11 (RBR genes) and this study (HECT genes). The table details the correlation coefficients for each pair of genes. In bold, significant comparisons (all of them with p<0.005 after Bonferroni’s correction, except the comparison *AT4g19670*/*UPL2*, which has p = 0.027).

## Discussion

In this work, by combining sequence analyses and structural data, the patterns of diversification of plant HECT ubiquitin ligases have been characterized. A first conclusion is that this family has followed a very conservative evolutionary pattern, in which a limited number of genes already present at the origin of the viridiplantae has been conserved intact in most lineages, with just a few lineage-specific gene duplications or gene losses (Figure 7 and Table 1). This has occurred despite a large number of genomic duplications in higher plants, meaning that HECT genes are extremely “resistant” to them, i. e. most genes produced after these duplications became subsequently lost [27]. This is in radical contrast with the results found in most other families of plant E3 proteins. For example, in some RBR ubiquitin ligases, a progressive increase in genes and several dramatic amplifications (e. g. in poaceae and brassicaceae species) have been detected [11]. Related results have been found for the ATL family of RING ubiquitin ligases [28], [29], the U-box family [30], [31] and for proteins involved in cullin E3 complexes, such as F-box proteins [32]–[38], Skp1 proteins [39], [40] and BTB proteins [41].

The general expression patterns described above (Figure 9) suggest that HECT proteins are acting in plants as part of the most fundamental cellular machinery. In good agreement, it has been described the involvement of two *Arabidopsis UPL* genes in basic processes, such as endoreplication (*UPL3*) and senescence (*UPL5*) [24], [25], [42]. From the evolutionary point of view, an interesting question is whether the resistance of HECT genes to be duplicated may be precisely related to them being broadly expressed, a hypothesis already suggested [11] for the set of housekeeping RBR genes which have been here compared with HECTs. If this is generally true for genes belonging to the ubiquitination machinery, we would expect plant species having a group of evolutionarily conservative genes (i. e. genes duplicated infrequently) and a second group that may rapidly amplify. Although data are still incomplete, this expectation fits well with what is hitherto known of plant ubiquitin ligases (Refs. [11], [28]–[41] and this study). In plants, there are strong forces that can select for gene multiplication, particularly responses to external challenges: interactions with pathogens as part of the plant innate immune response, answers to abiotic or biotic stress, etc. [43]–[47]. Notably, evidence for an involvement in innate immunity has already been found for members of all types of plant ubiquitin ligases except, precisely, the very conservative HECTs [45], [46]. A final consideration regarding the expression data is that finding a strong correlation of expression between totally unrelated RBR and HECT genes when many tissues and developmental times are analyzed (Table 2) does not actually require them to be directly connected from a functional point of view. It may be simply a byproduct of all them being housekeeping, i. e. a secondary effect of the intrinsic requirements for ubiquitination in each of those different samples.

Another general conclusion is that most HECT subfamilies today found in plants arose very early in eukaryotic evolution. The simplest hypothesis is that at least four genes were present before the split that gave rise to the animal and plant lineages. Based on sequences and common structures, all plant HECT subfamilies but Subfamily VI can be traced back in time to that split. This highlights even more conclusively that HECT genes are evolutionary conserved for long periods of time. The fact that these ancient genes encoded HECT E3s that already had different structures, with characteristic additional protein domains, hints to this early diversification being associated to distinct cellular roles already in early eukaryotic evolution.

A final significant conclusion is that the patterns of diversification of HECT genes in the transition from unicellularity to multicellularity are quite different in plants and animals. Before the advent of animal multicellularity, there were already no less than 14 HECT genes and five more appeared in the animal lineage just after the choanoflagellate/animal split [15]. In plants, on the contrary, the number of genes before the chlorophyte/strepthophyte split was much more limited, probably seven, and the transition to multicellularity barely increased that number (Figure 7). Another important difference is that many independent gene losses were detected in some animal lineages, (insects, nematodes, urochordates) leading to a much reduced number of HECT genes in those species [15]. This has not been observed in plants, in which only a few losses have been detected in some particular lineages (Figure 7 and Table 1). The functional reasons that may explain these differences remain unknown.

In summary, this study have not only shed new light on the potential of diversification of the HECT family of ubiquitin ligases but also opens interesting new views about how ubiquitin ligases as a whole are evolving in plants and how the ubiquitin system may be differently evolving in plants and animals. Further analyses of HECT E3s in other groups of organisms may contribute to our understanding of the long-term evolution of this class of proteins.

## Materials and Methods

I used as a starting point for this study the eukaryotic-wide database of 1081 aligned HECT domain sequences described in [15]. This database was updated (July 2012) by performing TBlastN analyses with multiple HECT sequences against the nr, wgs, htgs, gss, est and tsa databases of the National Center for Biotechnology Information (http://www.ncbi.nlm.nih.gov/). After eliminating duplicates and truncated sequences, I obtained a final dataset with 413 full-length viridiplantae sequences. These sequences were aligned with ClustalX 2.0.12 [48] and the alignment was manually corrected using the GeneDoc 2.7 sequence editor [49]. This final alignment, in fasta format, can be found in File S1. Additional searches for fragments of relevant genes which could change the evolutionary hypothesis for the origin and evolution of the family deduced from the main dataset were performed using also TblastN against the same databases indicated above. The few significant hits are described in the Results section and were incorporated in the description of the most parsimonious hypothesis for the diversification of the family shown in Figure 7. The comparisons between plant and animal HECT sequences described in Results involved adding to the main plant alignment all the animal and choanoflagellate sequences present in our databases. The final database containing plant, animal and choanoflagellate HECTs that was used to generate Figure 8 included 1031 sequences.

Phylogenetic analyses were similar to those used already in our previous papers, e. g. Ref. [15]. Three different methods of phylogenetic reconstruction were used. Neighbor-joining (NJ) and maxium-likelihood (ML) trees were obtained using MEGA 5 [50] and Maximum-parsimony (MP) trees were obtained using PAUP* 4.0, beta 10 version [51]. For NJ, Kimurás correction was used and sites with gaps were treated with the pairwise deletion option. Parameters for MP were as follows: 1) all sites included, gaps treated as unknown characters; 2) randomly generated trees used as seeds; 3) maximum number of trees saved equal to 100; and, 4) heuristic search using the nearest-neighbor interchange algorithm. Finally, for ML analyses, the BioNJ tree was used to start the iterative searches and the WAG model of amino acidic substitutions with uniform rates was selected. Gaps were also treated as unknown characters. The nearest-neighbor interchange routine was used to explore the landscape of ML trees. Bootstrap tests were performed to establish the reliability of the trees obtained. A total of 1000 replicates were generated for NJ analyses and 100 replicates were obtained for the MP and ML trees, which are much more computer intensive. MEGA5 was also used to edit and draw the trees in Figures 1, 2, 3, 4, 5, 6 and 8. The neighbor-joining trees from which those figures were built, which include all the names of the species and the accession numbers of the sequences, can be found in Newick tree format as Files S2, S3, S4, S5, S6. Structural searches were performed using the integrated tool InterProScan [52]. Microarray data for *Arabidopsis thaliana* developmental samples were obtained from [53]. Pearson’s correlation coefficients were calculated for the average values of the HECT and RBR genes and also, individually, for each pair of HECT/RBR comparison (see Table 2 and Results). Standard t test (assuming the null hypothesis H_o_: r = 0) were made to establish the significance of the values obtained. Bonferroni’s correction was applied to take into account that multiple tests were performed.

## Supporting Information

File S1 **Txt file, plant HECT alignment.**
(TXT)

File S2 **Txt file, NJ tree Subfamily I.**
(TXT)

File S3 **Txt file, NJ tree Subfamilies II and III.txt.**
(TXT)

File S4 **Txt file, NJ tree Subfamilies IV.txt.**
(TXT)

File S5 **Txt file, NJ tree subfamily V.txt.**
(TXT)

File S6 **Txt file, NJ tree subfamily VI.txt.**
(TXT)
